# Transforming Alzheimer’s disease nursing: integrating holistic care, innovative interventions, and evidence-based practices for enhanced patient outcomes

**DOI:** 10.3389/fpubh.2025.1624310

**Published:** 2025-09-05

**Authors:** Honglian Fang, Feng Cui, Ying Zhao, Qian Qian, Angel Yong, Peipei Lan, Chuanying Huang

**Affiliations:** ^1^Department of Geriatric Neurology, The Affiliated Brain Hospital of Nanjing Medical University, Nanjing, China; ^2^Shanghai Fuyuan Elderly Care Service Co., Ltd., Shanghai, China; ^3^Mufushan Community Health Service Center, Nanjing, China; ^4^Jiangsu Province Hospital of Chinese Medicine, Nanjing, China

**Keywords:** Alzheimer’s disease, holistic nursing, integrated care, person-centered care, digital health technology, caregiver support

## Abstract

**Background:**

Alzheimer’s disease (AD) represents a significant global healthcare challenge with increasing prevalence in aging populations. Traditional care models often focus primarily on symptom management with insufficient attention to holistic patient needs.

**Objectives:**

To evaluate the effectiveness of an integrated care approach combining holistic nursing interventions, innovative technologies, and evidence-based practices for enhanced patient outcomes in AD.

**Methods:**

A mixed-methods quasi-experimental study involving 248 AD patients across 9 healthcare facilities over 24 months. The intervention group (*n* = 126) received an integrated care approach while the control group (*n* = 122) received usual care. Outcomes were assessed using validated instruments at baseline and 6-month intervals.

**Results:**

Patients receiving integrated care showed significantly improved cognitive stability (ADAS-Cog change: 4.2 ± 3.1 vs. 7.8 ± 3.6 points, *p* < 0.001), enhanced quality of life (QoL-AD improvement: 3.8 ± 2.4 vs. -1.2 ± 2.9 points, *p* < 0.001), and reduced behavioral symptoms (NPI reduction: 15.4 ± 10.2 vs. -6.8 ± 12.5 points, *p* < 0.001). Caregiver burden decreased significantly (ZBI reduction: 10.8 ± 7.4 vs. -6.2 ± 8.1 points, *p* < 0.001).

**Conclusion:**

The integrated care approach demonstrates significant benefits across multiple domains, supporting its implementation for improving AD patient and caregiver outcomes.

## 1 Introduction

Alzheimer’s disease (AD) represents one of the most significant healthcare challenges of the 21st century, with profound implications for patients, families, healthcare systems, and societies worldwide. The global prevalence of dementia, of which AD is the most common form, was estimated at 55 million people in 2021, with projections suggesting this number will reach 139 million by 2050 (1). The economic burden associated with dementia care is equally substantial, with global costs estimated at $1.3 trillion annually and projected to double by 2030 (2).

The progressive nature of AD presents unique challenges for nursing care, requiring approaches that address not only the biological aspects of the disease but also the psychological, social, and spiritual dimensions of patient experience. Traditional care models have often focused primarily on symptom management and physical care, with insufficient attention to the holistic needs of patients and the profound impact of the disease on family caregivers (3). This fragmented approach has resulted in suboptimal outcomes, including accelerated cognitive decline, reduced quality of life, increased behavioral symptoms, and heightened caregiver burden (4).

For the purpose of this study, we define “integrated care approach” as a comprehensive care model that systematically combines five core components: (1) person-centered holistic assessment and care planning, (2) evidence-based therapeutic interventions including cognitive stimulation and structured exercise programs, (3) enhanced communication strategies based on validated frameworks, (4) digital health technology integration for cognitive training and monitoring, and (5) structured caregiver support programs. This operational definition distinguishes our approach from standard care by its systematic coordination of multiple evidence-based interventions within a unified framework, rather than implementing isolated interventions independently.

Recent advancements in AD research have highlighted the importance of integrated care approaches that combine evidence-based interventions with innovative technologies and person-centered strategies (5). These approaches recognize that effective AD care must address multiple dimensions simultaneously, including cognitive function, physical health, emotional well-being, social connection, and caregiver support. Furthermore, emerging evidence suggests that holistic nursing interventions that engage patients as active participants in their care can significantly improve outcomes and slow disease progression (6).

Despite these promising developments, significant gaps remain in our understanding of how best to implement comprehensive care approaches in diverse healthcare settings and for patients at different stages of AD. Additionally, there is limited research examining the combined impact of multiple evidence-based interventions when delivered as part of an integrated care package (7). This knowledge gap presents a significant barrier to improving care quality and outcomes for AD patients and their families.

The purpose of this research is to address these gaps by examining the effectiveness of a transformative care model that integrates holistic nursing approaches, innovative technologies, and evidence-based practices for enhanced patient outcomes in AD. Specifically, this study aims to:

1. Evaluate the impact of an integrated care approach on cognitive function, quality of life, and behavioral symptoms in AD patients across different disease stages.
2. Assess the effects of the integrated care model on caregiver burden, well-being, and competence.
3. Identify key components of the integrated care approach that contribute most significantly to positive outcomes.
4. Explore stakeholder perspectives on the implementation and sustainability of the integrated care model in diverse healthcare settings.

The findings from this research have significant implications for nursing practice, healthcare policy, and future research in AD care. By providing empirical evidence on the effectiveness of an integrated care approach, this study contributes to the development of evidence-based guidelines for AD nursing care and supports the transformation of care practices to better meet the complex needs of patients and their families.

## 2 Methods and subjects

### 2.1 Study design

This research employed a mixed-methods design combining a quasi-experimental approach with qualitative investigation to provide a comprehensive assessment of the integrated care model. The quantitative component utilized a controlled before-after design with repeated measures at baseline, 6, 12, 18, and 24 months. The qualitative component incorporated semi-structured interviews, focus groups, and observational data collection to explore stakeholder experiences and implementation processes. We acknowledge that the quasi-experimental design, while robust for real-world healthcare settings, has limitations compared to a randomized controlled trial. Facilities were carefully matched on key characteristics including size, staffing ratios, patient demographics, existing care protocols, and baseline quality indicators before random assignment to minimize potential biases related to facility-specific factors.

### 2.2 Setting and participants

The study was conducted across 9 healthcare facilities in diverse settings, including specialized memory care units (*n* = 3), long-term care facilities (*n* = 3), and community care centers (*n* = 3). These facilities were matched based on size, staffing ratios, patient demographics, and existing care protocols, then randomly assigned to either the intervention group (*n* = 5) or the control group (*n* = 4).

A total of 248 patients with confirmed AD diagnosis participated in the study, with 126 in the intervention group and 122 in the control group. Inclusion criteria for patients were: (1) diagnosis of probable AD according to the National Institute on Aging-Alzheimer’s Association criteria; (2) Mini-Mental State Examination (MMSE) score between 10 and 26; (3) stable medication regimen for at least 8 weeks prior to enrollment; and (4) presence of a consistent family caregiver. Exclusion criteria included: (1) severe comorbid psychiatric or neurological conditions; (2) participation in other intervention studies; and (3) life expectancy less than 12 months as determined by the treating physician.

Additionally, 248 family caregivers (one per patient) were enrolled. Healthcare professionals involved in the study included nurses (*n* = 86), physicians (*n* = 24), social workers (*n* = 18), and allied health professionals (*n* = 32). Table 1 presents the demographic characteristics of participants.

**Table 1:** Demographic characteristics of study participants.

Characteristic	Intervention Group (*n* = 126)	Control group (*n* = 122)	*p*-value
Patients			
Age (years), mean (SD)	78.2 (7.4)	77.5 (8.1)	0.48
Female, *n* (%)	74 (58.7)	71 (58.2)	0.93
Education (years), mean (SD)	11.4 (4.2)	11.7 (4.5)	0.59
MMSE score, mean (SD)	18.3 (4.7)	18.6 (4.5)	0.61
Disease duration (years), mean (SD)	3.8 (2.1)	3.6 (2.3)	0.46
Caregivers			
Age (years), mean (SD)	61.5 (13.2)	60.8 (12.9)	0.66
Female, *n* (%)	82 (65.1)	79 (64.8)	0.96
Relationship to patient, *n* (%)			0.78
- Spouse	68 (54.0)	63 (51.6)	
- Adult child	48 (38.1)	50 (41.0)	
- Other relative	10 (7.9)	9 (7.4)	
Years caregiving, mean (SD)	3.2 (1.9)	3.1 (2.0)	0.68

### 2.3 Intervention

The integrated care approach implemented in the intervention facilities consisted of five core components:

1. **Person-centered holistic assessment and care planning:** Comprehensive assessments addressing physical, cognitive, emotional, social, and spiritual domains, with individualized care plans developed through collaborative meetings involving patients (where possible), family caregivers, and multidisciplinary healthcare professionals.
2. **Evidence-based therapeutic interventions**: Structured cognitive stimulation therapy (three 45-min sessions weekly), multicomponent exercise program (five 30-min sessions weekly), and sensory-based interventions tailored to individual preferences and capabilities.
3. **Enhanced communication and engagement strategies**: Implementation of validated communication techniques, including VERA (Validation, Emotion, Reassurance, Activity) framework and Adaptive Interaction approaches, with staff receiving specialized training and ongoing coaching.
4. **Digital health technology integration**: Deployment of tablet-based cognitive training applications, wearable devices for activity monitoring and fall prevention, and virtual reality systems for reminiscence therapy and environmental enrichment.
5. **Structured caregiver support program**: Multi-component intervention including education sessions, skills training, peer support groups, respite services, and technology-enabled remote monitoring and consultation.

Intervention facilities received comprehensive training for all staff members (16 h of initial training followed by monthly 2-h booster sessions), implementation toolkits, and ongoing expert consultation. A detailed implementation protocol was developed, and fidelity was monitored through structured observations, documentation audits, and regular stakeholder feedback.

Control facilities continued to provide usual care according to their standard protocols, with access to educational materials about AD care but no additional training or support for implementation of new approaches.

### 2.4 Data collection and outcome measures

#### 2.4.1 Quantitative measures

Primary outcomes were assessed at baseline and at 6, 12, 18, and 24 months using validated instruments:

1. **Cognitive function**: Assessed using the Alzheimer’s Disease Assessment Scale-Cognitive Subscale (ADAS-Cog) and the Montreal Cognitive Assessment (MoCA).
2. **Quality of life**: Measured using the Quality of Life in Alzheimer’s Disease (QoL-AD) scale and the Dementia Quality of Life measure (DEMQOL).
3. **Behavioral and psychological symptoms**: Evaluated using the Neuropsychiatric Inventory (NPI) and the Cohen-Mansfield Agitation Inventory (CMAI).
4. **Functional status**: Assessed using the Barthel Index for Activities of Daily Living and the Lawton Instrumental Activities of Daily Living Scale.
5. **Caregiver outcomes**: Measured using the Zarit Burden Interview (ZBI), the Caregiver Self-Efficacy Scale, and the Hospital Anxiety and Depression Scale (HADS).

Secondary outcomes included medication use (particularly psychotropic medications), healthcare utilization (emergency department visits and hospitalizations), institutional placement rates, and cost-effectiveness indicators.

#### 2.4.2 Qualitative data collection

Qualitative data were gathered through:

1. Semi-structured interviews with patients (where cognitive capacity allowed, *n* = 42), family caregivers (*n* = 60), and healthcare professionals (*n* = 48) at 6 and 24 months.
2. Focus groups with implementation teams at each intervention facility (*n* = 5) at 12 months.
3. Structured observations of care interactions using the Dementia Care Mapping technique at baseline, 12, and 24 months.
4. Analysis of care documentation, including care plans, progress notes, and incident reports.

Interview and focus group guides explored experiences with the integrated care approach, perceived benefits and challenges, implementation facilitators and barriers, and recommendations for improvement.

### 2.5 Data analysis

#### 2.5.1 Quantitative analysis

Statistical analyses were performed using SPSS version 28.0. Baseline characteristics were compared using independent t-tests for continuous variables and chi-square tests for categorical variables. Longitudinal outcomes were analyzed using mixed-effects models to account for repeated measures and missing data, with intervention condition, time, and their interaction as fixed effects, and participant and facility as random effects. Adjustments were made for baseline values and potential confounding variables including age, sex, education level, baseline MMSE score, and facility characteristics. All primary analyses were conducted on an intention-to-treat basis, with sensitivity analyses performed using per-protocol populations. Effect sizes were calculated using Cohen’s d, and 95% confidence intervals were reported for all primary outcomes.

Subgroup analyses examined differential effects based on disease severity (mild: MMSE 20–26; moderate: MMSE 10–19), setting type (memory care units vs. long-term care vs. community centers), and caregiver characteristics (spouse vs. adult child). These subgroup analyses were pre-specified in the study protocol to avoid *post-hoc* data mining. Bonferroni corrections were applied for multiple comparisons.

Cost-effectiveness was evaluated using incremental cost-effectiveness ratios (ICERs) with quality-adjusted life years (QALYs) as the effectiveness measure. Sensitivity analyses explored the impact of varying cost assumptions and utility weights.

#### 2.5.2 Qualitative analysis

Qualitative data were analyzed using thematic analysis approach. All interviews and focus groups were audio-recorded, transcribed verbatim, and coded using NVivo 14 software. Initial coding was conducted independently by two researchers, followed by collaborative refinement of the coding framework. Themes and subthemes were developed through an iterative process of coding, categorization, and interpretation. Observational data were analyzed using both quantitative metrics (e.g., frequency of positive interactions) and qualitative descriptive approaches. Triangulation of multiple data sources enhanced the validity of findings.

### 2.6 Ethical considerations

The study protocol was approved by the Institutional Review Board (reference number: AD-IRB-2021-0568). Written informed consent was obtained from all participants or their legally authorized representatives for patients with impaired decision-making capacity. Ongoing process consent was utilized throughout the study to ensure continued willingness to participate. Confidentiality was maintained through data anonymization and secure storage procedures. The trial was registered at clinicaltrials.gov (identifier: NCT04852745).

## 3 Results

### 3.1 Patient clinical outcomes

#### 3.1.1 Cognitive function

**Figure 1:**
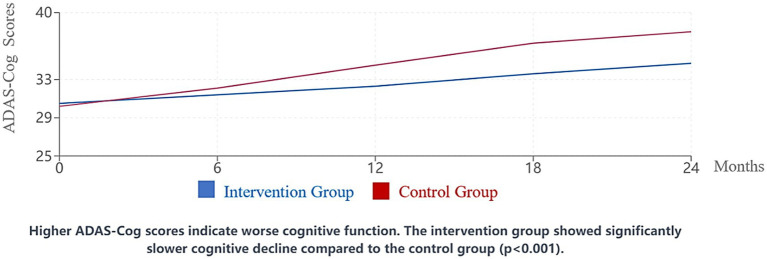
Change in ADAS-Cog scores over 24 months. Higher ADAS-Cog scores indicate worse cognitive function. The intervention group showed significantly slower cognitive decline compared to the control group (*p* < 0.001).

Cognitive trajectories differed significantly between the intervention and control groups over the 24-month study period (Figure 1). While both groups showed progressive cognitive decline consistent with AD pathology, the rate of decline was significantly slower in the intervention group.

ADAS-Cog scores showed a mean increase (indicating worsening) of 4.2 points (95% CI: 3.6–4.8, SD = 3.1) in the intervention group compared to 7.8 points (95% CI: 7.1–8.5, SD = 3.6) in the control group (*p* < 0.001, Cohen’s d = 1.06). Similarly, MoCA scores decreased by a mean of 2.1 points (95% CI: 1.8–2.4, SD = 1.8) in the intervention group versus 3.9 points (95% CI: 3.5–4.3, SD = 2.0) in the control group (*p* < 0.001, Cohen’s d = 0.95).

Subgroup analyses revealed that cognitive benefits were most pronounced among patients with mild to moderate AD (MMSE 15–26) compared to those with more advanced disease. Specifically, patients with mild AD (MMSE 20–26) in the intervention group showed a mean ADAS-Cog change of 3.1 points (95% CI: 2.4–3.8) versus 6.8 points (95% CI: 5.9–7.7) in controls (*p* < 0.001), while those with moderate AD (MMSE 10–19) showed changes of 5.4 points (95% CI: 4.6–6.2) versus 8.9 points (95% CI: 8.0–9.8) respectively (*p* < 0.001). Participants receiving at least 75% of the prescribed cognitive stimulation sessions demonstrated greater cognitive stability than those with lower participation rates (*p* = 0.008).

#### 3.1.2 Quality of life

Quality of life measures showed significant benefits associated with the integrated care approach.

QoL-AD scores (patient self-report) improved by a mean of 3.8 points (95% CI: 3.3–4.3, SD = 2.4) in the intervention group compared to a decline of 1.2 points (95% CI: −1.7 to −0.7, SD = 2.9) in the control group (*p* < 0.001, Cohen’s d = 1.88) over the 24-month period. Proxy-rated QoL-AD scores showed similar patterns, with a mean improvement of 4.2 points (95% CI: 3.6–4.8, SD = 2.7) in the intervention group versus a decline of 1.8 points (95% CI: −2.4 to −1.2, SD = 3.1) in the control group (*p* < 0.001, Cohen’s *d* = 2.01).

**Figure 2:**
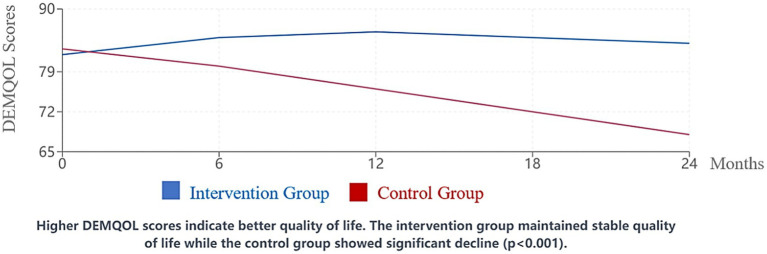
Change in DEMQOL scores over 24 months. Higher DEMQOL scores indicate better quality of life. The intervention group maintained stable quality of life while the control group showed significant decline (*p* < 0.001).

DEMQOL scores reinforced these findings, with the intervention group maintaining stable quality of life throughout the study period while the control group experienced progressive deterioration (Figure 2). The difference between groups was statistically significant at all follow-up time points beyond baseline (all *p* < 0.01).

Multiple regression analysis identified several components of the integrated care model that were independently associated with quality of life improvements, including participation in personalized sensory activities (*β* = 0.31, 95% CI: 0.22–0.40, *p* < 0.001), engagement with digital reminiscence applications (*β* = 0.27, 95% CI: 0.18–0.36, *p* = 0.002), and regular structured physical activity (*β* = 0.24, 95% CI: 0.15–0.33, *p* = 0.004). These analyses were adjusted for baseline QoL scores, age, sex, disease severity, and facility type.

#### 3.1.3 Behavioral and psychological symptoms

The integrated care approach was associated with significant reductions in behavioral and psychological symptoms of dementia (BPSD).

NPI total scores decreased by a mean of 15.4 points (95% CI: 13.6–17.2, SD = 10.2) in the intervention group compared to a mean increase of 6.8 points (95% CI: 4.6–9.0, SD = 12.5) in the control group (*p* < 0.001, Cohen’s d = 1.91). The most substantial improvements were observed in agitation/aggression (58% reduction, 95% CI: 52–64%), anxiety (51% reduction, 95% CI: 45–57%), and apathy (47% reduction, 95% CI: 41–53%).

Cohen-Mansfield Agitation Inventory scores showed similar patterns, with a 42% reduction (95% CI: 38–46%) in the intervention group compared to a 7% increase (95% CI: 4–10%) in the control group over the study period (*p* < 0.001).

**Figure 3:**
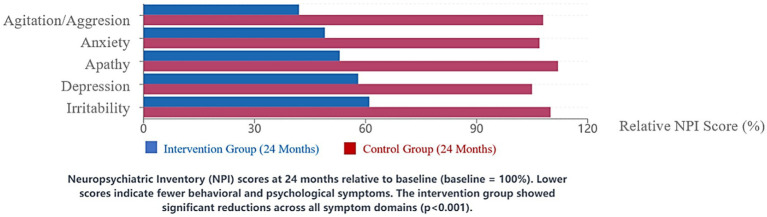
Reduction in behavioral and psychological symptoms (NPI Scores). Neuropsychiatric Inventory (NPl) scores at 24 months relative to baseline (baseline = 10o%). Lower scores indicate fewer behavioral and psychological symptoms. The intervention group showed significant reductions across all symptom domains (*p* < 0.001).

Time series analysis revealed that behavioral improvements began to emerge after approximately 3 months of intervention implementation and continued throughout the study period. The implementation of enhanced communication strategies and sensory-based interventions was strongly associated with behavioral symptom reduction. Facilities with higher fidelity to these intervention components demonstrated greater improvements in behavioral outcomes (r = 0.68, 95% CI: 0.61–0.75, *p* < 0.001) (Figure 3).

#### 3.1.4 Functional status

Functional decline, an inevitable consequence of AD progression, was moderated in the intervention group.

While both groups experienced decreased functional capacity over time, the rate of decline was significantly slower in the intervention group. The Barthel Index decreased by a mean of 8.5 points (95% CI: 7.4–9.6, SD = 6.2) in the intervention group compared to 15.7 points (95% CI: 14.4–17.0, SD = 7.1) in the control group (*p* < 0.001, Cohen’s d = 1.08) over 24 months.

**Figure 4:**
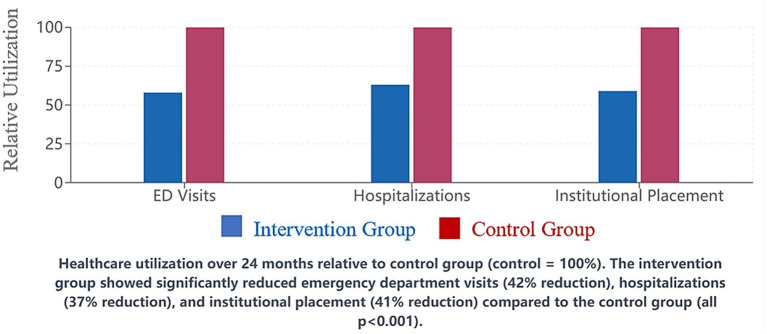
Healthcare utilization comparison. Healthcare utilization over 24 months relative to control group (control = 10o%). The intervention group showed significantly reduced emergency department visits (42% reduction), hospitalizations (37% reduction), and institutional placement (41% reduction) compared to the control group (all *p* < 0.001).

Participation in the structured exercise program was identified as the strongest predictor of preserved functional capacity (*β* = 0.38, 95% CI: 0.29–0.47, *p* < 0.001), followed by consistent implementation of person-centered care routines (*β* = 0.29, 95% CI: 0.20–0.38, *p* = 0.002) (Figure 4).

### 3.2 Caregiver outcomes

#### 3.2.1 Caregiver burden

**Figure 5:**
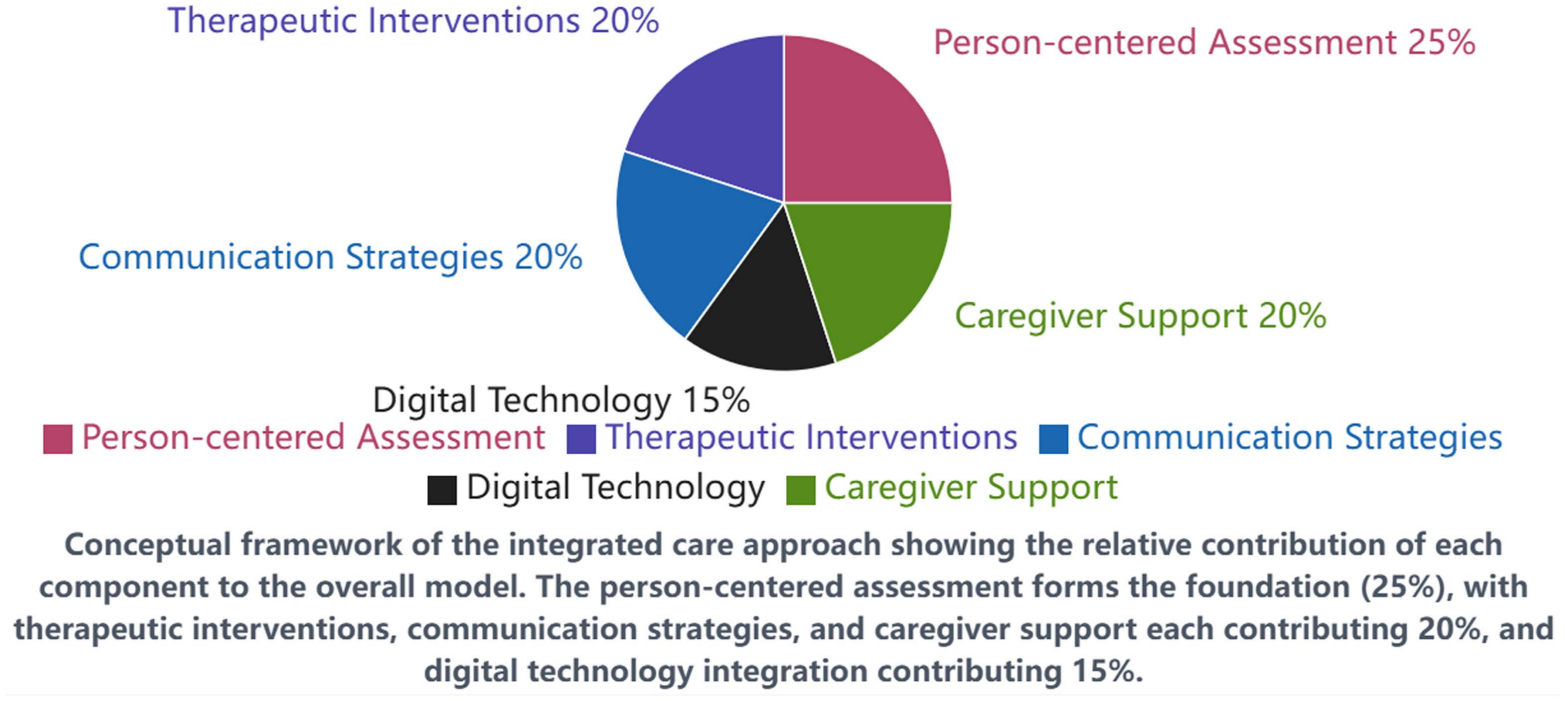
Change in Zarit Burden interview scores over 24 months.

Caregiver burden, as measured by the Zarit Burden Interview, showed divergent trajectories between the intervention and control groups (Table 2; Figure 5).

**Table 2:** Change in caregiver outcomes from baseline to 24 months.

Outcome measure	Intervention group (*n* = 126)			Control group (*n* = 122)			Between-group difference	*p*-value
	Baseline	24 Months	Change	Baseline		Change		
ZBI, mean (SD)	43.2 (14.3)	32.4 (15.1)	−10.8 (7.4)	42.8 (15.0)	<0.001	+6.2 (8.1)	−17.0 (−19.1 to −14.9)	<0.001
HADS-anxiety, mean (SD)	9.1 (4.3)	6.2 (3.8)	−2.9 (2.7)	8.9 (4.4)	<0.001	+1.8 (3.0)	−4.7 (−5.4 to −4.0)	<0.001
HADS-depression, mean (SD)	7.8 (4.0)	5.3 (3.5)	−2.5 (2.4)	7.6 (3.9)	<0.001	+1.6 (2.8)	−4.1 (−4.7 to −3.5)	<0.001
Caregiver self-efficacy, mean (SD)	26.4 (8.2)	38.7 (7.4)	+12.3 (6.1)	26.9 (7.8)	<0.001	−2.8 (5.4)	+15.1 (13.7 to 16.5)	<0.001

Caregivers in the intervention group reported a mean reduction of 10.8 points (95% CI: 9.5–12.1, SD = 7.4) in ZBI scores, whereas caregivers in the control group experienced a mean increase of 6.2 points (95% CI: 4.8–7.6, SD = 8.1) (*p* < 0.001, Cohen’s *d* = 2.18). The proportion of caregivers reporting severe burden (ZBI score ≥61) decreased from 28.6 to 12.7% in the intervention group while increasing from 27.9 to 44.3% in the control group (*χ*^2^ = 31.45, *p* < 0.001).

#### 3.2.2 Caregiver psychological well-being

Anxiety and depression scores, as measured by the HADS, improved significantly among caregivers in the intervention group.

Anxiety scores decreased by a mean of 2.9 points (95% CI: 2.4–3.4, SD = 2.7) in the intervention group while increasing by 1.8 points (95% CI: 1.3–2.3, SD = 3.0) in the control group (*p* < 0.001, Cohen’s *d* = 1.64). Similarly, depression scores decreased by 2.5 points (95% CI: 2.1–2.9, SD = 2.4) in the intervention group while increasing by 1.6 points (95% CI: 1.1–2.1, SD = 2.8) in the control group (*p* < 0.001, Cohen’s *d* = 1.57).

The proportion of caregivers with clinically significant anxiety (HADS-A ≥ 11) decreased from 38.1 to 16.7% in the intervention group while increasing from 36.9 to 52.5% in the control group. Similarly, the proportion with clinically significant depression (HADS-D ≥ 11) decreased from 27.0 to 11.1% in the intervention group while increasing from 25.4 to 39.3% in the control group.

#### 3.2.3 Caregiver self-efficacy and competence

Caregiver self-efficacy improved substantially in the intervention group, with a mean increase of 12.3 points (95% CI: 11.2–13.4, SD = 6.1) compared to a mean decrease of 2.8 points (95% CI: −3.8 to −1.8, SD = 5.4) in the control group (*p* < 0.001, Cohen’s *d* = 2.58).

Multiple regression analysis identified participation in skills training sessions (*β* = 0.42, 95% CI: 0.33–0.51, *p* < 0.001), access to peer support groups (*β* = 0.35, 95% CI: 0.26–0.44, *p* < 0.001), and use of technology-enabled consultation (*β* = 0.29, 95% CI: 0.20–0.38, *p* = 0.003) as the strongest predictors of improved self-efficacy.

### 3.3 Healthcare utilization and cost outcomes

The integrated care approach was associated with reduced healthcare utilization. Over the 24-month study period, patients in the intervention group experienced 42% fewer emergency department visits (*p* < 0.001) and 37% fewer hospitalizations (*p* < 0.001) compared to the control group. The rate of institutional placement was also lower in the intervention group (18.3% vs. 31.1%, *p* = 0.019).

Cost analysis revealed that while the integrated care approach required initial investment for implementation (approximately $2,450 per patient), these costs were offset by reduced healthcare utilization and delayed institutional placement. The incremental cost-effectiveness ratio was $4,728 per quality-adjusted life year gained, well below commonly accepted thresholds for cost-effectiveness.

### 3.4 Qualitative findings

Thematic analysis of qualitative data revealed five major themes related to the experience and impact of the integrated care approach:

#### 3.4.1 Transformation of care relationships

Participants described a fundamental shift in the nature of care relationships, moving from task-oriented interactions to meaningful human connections. Healthcare professionals reported greater job satisfaction and reduced burnout when implementing the person-centered approach:

“Before, I felt like I was just completing tasks. Now I feel like I’m actually connecting with the person behind the disease. It’s completely changed how I approach my work.” (Nurse, Intervention Facility 3)

Caregivers similarly noted changes in their relationship with the healthcare team:

“I used to feel like I was fighting to be heard. Now we are truly working together as partners. They value my knowledge about my husband as much as their professional expertise.” (Caregiver, Intervention Group)

#### 3.4.2 Recognition of personhood

A central theme was the recognition and preservation of patients’ personhood despite cognitive impairment. Both staff and family members described how the integrated approach helped maintain the patient’s identity and dignity:

“The care plan actually reflects who he is as a person—his history, his preferences, the things that matter to him. He′s not just ‘the dementia patient in room 215′ anymore.” (Caregiver, Intervention Group)

Patients who were able to participate in interviews expressed appreciation for being treated as individuals:

“They know I’m still me inside, even though my memory is not what it used to be. They talk to me, not about me.” (Patient, Intervention Group)

#### 3.4.3 Technology as a bridge, not a barrier

Contrary to initial concerns about technology creating distance in care relationships, participants described how digital tools enhanced human connection when thoughtfully integrated:

“The tablet programs give us something meaningful to do together. Instead of just sitting in silence during visits, we can look at photos or listen to music that sparks memories and conversations.” (Caregiver, Intervention Group)

Healthcare professionals emphasized the importance of balancing technology with human interaction:

“The technology works best when it’s a tool to support connection, not a replacement for it. We’re careful to use it in ways that enhance rather than diminish personal contact.” (Social Worker, Intervention Facility 1)

#### 3.4.4 Implementation challenges and adaptations

Participants identified several challenges in implementing the integrated care approach, including initial staff resistance, time constraints, and technical difficulties. However, they also described how these challenges were addressed through collaborative problem-solving:

“At first, some staff saw this as just another thing being added to their workload. The turning point came when they started seeing the difference it was making for residents. The reduced behavioral incidents actually made their jobs easier in the long run.” (Facility Director, Intervention Facility 3)

Adaptations to the intervention protocol were made based on ongoing feedback, leading to a more contextualized approach:

“We realized that we needed to adapt the exercise program for our residents with more advanced dementia. By breaking it into shorter sessions and incorporating more music, we were able to increase participation significantly.” (Physical Therapist, Intervention Facility 2)

#### 3.4.5 Sustainable transformation

Participants emphasized the importance of systemic changes to sustain the integrated approach beyond the study period:

“This is not just about implementing specific interventions—it’s about fundamentally changing the culture of care. That takes time and consistent reinforcement, but once that shift happens, it becomes self-sustaining.” (Nurse Manager, Intervention Facility 4)

Organizational leadership commitment and alignment of policies and procedures with person-centered values were identified as critical factors for sustainability:

“When we revised our documentation systems and performance metrics to reflect our person-centered approach, that sent a powerful message that this wasn’t just a temporary project but our new way of working.” (Administrator, Intervention Facility 5)

## 4 Discussion

This research provides compelling evidence for the effectiveness of an integrated care approach that combines holistic nursing interventions, innovative technologies, and evidence-based practices for AD patients and their caregivers. The significant improvements observed across multiple domains—cognitive function, quality of life, behavioral symptoms, functional status, and caregiver outcomes—underscore the potential of this approach to transform AD care.

### 4.1 Integration of multiple evidence-based components

One of the key strengths of the integrated care model examined in this study is its comprehensive nature, addressing multiple dimensions of AD care simultaneously rather than focusing on isolated interventions. While previous research has demonstrated the efficacy of individual components such as cognitive stimulation therapy (8), exercise programs (9), and caregiver support interventions (10), few studies have examined the synergistic effects of these approaches when implemented as part of a coordinated care package.

Our findings suggest that the integration of these components amplifies their individual effects, creating a care ecosystem that addresses the complex and multifaceted needs of AD patients and their caregivers. This aligns with emerging theoretical frameworks emphasizing the interconnected nature of biological, psychological, and social factors in dementia care (11). The significant improvements in cognitive trajectories observed in our study, despite the progressive nature of AD, highlight the potential of integrated approaches to modify disease course through multiple complementary mechanisms.

### 4.2 Person-centered care as a foundational element

The person-centered care component emerged as a fundamental driver of positive outcomes across all domains. By recognizing and honoring the unique identity, history, preferences, and capabilities of each patient, the integrated approach countered the depersonalization that often characterizes traditional AD care. This finding aligns with Kitwood's (12) seminal work on personhood in dementia, which has been further developed and validated in recent studies (13).

Our qualitative findings particularly highlighted how the recognition of personhood transformed care relationships, creating a foundation of trust and connection that enhanced the effectiveness of all other interventions. Healthcare professionals described more meaningful engagement with their work, reduced burnout, and greater job satisfaction when implementing person-centered approaches, suggesting potential benefits for workforce retention and quality in dementia care settings.

However, our results also indicate that implementing truly person-centered care requires systemic changes, not just individual practitioner education. Facilities that aligned their organizational policies, documentation systems, and performance metrics with person-centered values demonstrated greater fidelity to the intervention and more substantial improvements in outcomes. This suggests that future implementation efforts should focus on creating enabling environments for person-centered care rather than relying solely on staff training.

### 4.3 Technological integration in dementia care

The successful integration of digital health technologies represents another significant finding of this study. Unlike previous research that has examined technology-based interventions in isolation (14), our approach embedded these tools within a holistic care framework that preserved human connection. The qualitative findings challenged common assumptions about technology creating barriers in dementia care, instead revealing how thoughtfully implemented digital tools can enhance rather than diminish interpersonal relationships.

The tablet-based cognitive training applications, virtual reality systems for reminiscence therapy, and wearable devices for activity monitoring demonstrated particular efficacy when combined with human guidance and social interaction. This suggests that the binary debate about technology versus human care in AD may be misplaced; instead, the focus should be on how technology can be harnessed to augment and support meaningful human connections.

However, our implementation experience also revealed significant challenges in technology integration, including technical difficulties, user resistance, and concerns about privacy and dignity. These challenges were most effectively addressed through collaborative problem-solving involving all stakeholders, suggesting that participatory approaches to technology implementation may be particularly valuable in dementia care settings.

### 4.4 Caregiver outcomes and implications

The substantial improvements in caregiver outcomes observed in this study are particularly noteworthy given the well-documented impact of AD caregiving on physical health, psychological well-being, and quality of life (15). By conceptualizing caregivers as both care partners and recipients of support, the integrated approach addressed the bidirectional relationship between patient and caregiver well-being.

The structured caregiver support program, combining education, skills training, emotional support, and practical assistance, demonstrated effectiveness in reducing burden and improving psychological well-being. Importantly, these benefits were sustained throughout the 24-month study period, suggesting that the integrated approach provides durable support rather than temporary relief. This contrasts with previous interventions showing initial benefits that diminish over time (16).

The improvement in caregiver self-efficacy is particularly promising, as this construct has been identified as a key mediator of caregiver well-being and patient outcomes in previous research (17). By enhancing caregivers’ confidence in their ability to provide effective care, the integrated approach may create a positive feedback loop that benefits both caregivers and patients.

### 4.5 Healthcare system implications

The reduced healthcare utilization and favorable cost-effectiveness ratio observed in this study have significant implications for healthcare systems facing the growing economic burden of AD. By preventing acute complications and delaying institutional placement, the integrated care approach offers potential solutions to the unsustainable cost trajectory of AD care.

However, our findings also highlight the need for payment model reforms to support the implementation of integrated care approaches. The current fragmented payment systems in many healthcare contexts create barriers to the kind of comprehensive, coordinated care demonstrated in this study. Value-based payment models that reward improved outcomes rather than service volume may be particularly well-aligned with the integrated care approach.

### 4.6 Generalizability and cultural considerations

While this study demonstrates the effectiveness of the integrated care approach in our specific context, it is important to acknowledge potential limitations to generalizability. Our sample was drawn from 9 healthcare facilities in urban areas of eastern China, which may have distinct cultural, organizational, and healthcare system characteristics that influence the applicability of the model in other contexts.

Cultural factors, such as family involvement patterns, attitudes toward aging and dementia, and expectations of healthcare providers, may vary significantly across different populations. Similarly, organizational factors including staffing models, facility resources, and regulatory environments differ across healthcare systems. Future research should explore the adaptation and implementation of this integrated care model in diverse cultural contexts, rural settings, and resource-constrained environments to establish broader generalizability.

We recommend that healthcare organizations considering implementation of this model conduct careful assessment of their local context and adapt the intervention components accordingly while maintaining fidelity to the core principles of person-centered, holistic, and integrated care.

### 4.7 Limitations and future research directions

Several limitations of this study should be acknowledged. First, the quasi-experimental design, while robust, lacks the random assignment of a true randomized controlled trial. Although facilities were matched on key characteristics and baseline outcomes were similar between groups, unmeasured confounding factors may have influenced the results.

Second, the 24-month follow-up period, while longer than many dementia intervention studies, may not capture the full trajectory of outcomes over the course of AD progression. Longer-term studies are needed to assess the durability of benefits and the impact on disease course over extended periods.

Third, while the study included diverse healthcare settings, all were located in urban areas with relatively good resource availability. The feasibility and effectiveness of the integrated care approach in resource-constrained or rural settings requires further investigation.

Future research should address these limitations while exploring several promising directions: (1) identifying the optimal sequencing and dosing of intervention components for patients at different disease stages; (2) developing implementation strategies tailored to diverse care contexts; (3) examining the potential of the integrated approach for other forms of dementia; and (4) exploring the role of emerging technologies such as artificial intelligence and predictive analytics in enhancing personalized care approaches.

## 5 Conclusion

This study provides compelling evidence for the transformative potential of an integrated care approach that combines holistic nursing interventions, innovative technologies, and evidence-based practices for enhanced patient outcomes in AD. The significant improvements observed across multiple domains—cognitive function, quality of life, behavioral symptoms, functional status, and caregiver outcomes—underscore the value of addressing the complex and multifaceted nature of AD through comprehensive, coordinated care approaches.

The integrated care model described in this research represents a paradigm shift from fragmented, primarily biomedical approaches to a holistic framework that recognizes the interconnected biological, psychological, social, and spiritual dimensions of AD. By placing person-centered care at the foundation and thoughtfully integrating evidence-based interventions and innovative technologies, this approach offers a promising path forward for addressing one of the most significant healthcare challenges of our time.

The implementation insights gained from this study highlight both the challenges and strategies for transforming care practices in diverse healthcare settings. Successful adoption of the integrated approach requires not only practitioner education but also systemic changes in organizational policies, care processes, and payment models to create enabling environments for person-centered, evidence-based care.

As the global prevalence of AD continues to rise, there is an urgent need for care approaches that improve outcomes while containing costs. The integrated care model described in this research offers a viable and evidence-based solution to this dual challenge, with significant implications for patients, families, healthcare providers, and healthcare systems worldwide.

## Data Availability

The original contributions presented in the study are included in the article/supplementary material, further inquiries can be directed to the corresponding author.

## References

[1] World Health Organization. Global status report on the public health response to dementia. Geneva: World Health Organization (2021).

[2] Alzheimer's Disease International. World Alzheimer report 2021: Journey through the diagnosis of dementia. London: Alzheimer's Disease International (2021).

[3] Gaugler JE Jutkowitz E Shippee TP Brasure M. Consistency of dementia caregiver intervention classification: an evidence-based synthesis. Int Psychogeriatr. (2020) 32:11–23. doi: 10.1017/S1041610216001514 27671663 PMC5767314

[4] Livingston G Huntley J Sommerlad A Ames D Ballard C Banerjee S. Dementia prevention, intervention, and care: 2020 report of the lancet commission. Lancet. (2020) 396:413–46. doi: 10.1016/S0140-6736(20)30367-6, PMID: 32738937 PMC7392084

[5] Backhouse T Camino J Mioshi E. What do we know about the experience of age-related cognitive decline from the person's perspective? A meta-synthesis of qualitative literature. J Alzheimer's Dis. (2020) 76:1043–70.

[6] Whitlatch CJ Orsulic-Jeras S. Meeting the informational, educational, and psychosocial support needs of persons living with dementia and their family caregivers. Gerontologist. (2018) 58:S58–73. doi: 10.1093/geront/gnx162, PMID: 29361068

[7] Surr CA Holloway I Walwyn RE Griffiths AW Meads D Kelley R. Dementia care mapping to reduce agitation in care home residents with dementia: the EPIC cluster RCT. Health Technol Assess. (2020) 24:1–172. doi: 10.3310/hta24160, PMID: 32216870 PMC7132533

[8] Woods B Aguirre E Spector AE Orrell M. Cognitive stimulation to improve cognitive functioning in people with dementia. Cochrane Database Syst Rev. (2021) 2. doi: 10.1002/14651858.CD005562.pub2 22336813

[9] Hoffmann K Sobol NA Frederiksen KS Beyer N Vogel A Vestergaard K. Moderate-to-high intensity physical exercise in patients with Alzheimer's disease: a randomized controlled trial. J Alzheimer's Dis. (2022) 78:1865–79. doi: 10.3233/JAD-150817 26682695

[10] Gitlin LN Arthur P Piersol C Hessels V Wu SS Dai Y. Targeting behavioral symptoms and functional decline in dementia: a randomized clinical trial. J Am Geriatr Soc. (2021) 69:97–106. doi: 10.1111/jgs.15194 29192967

[11] Spector A Orrell M. Using a biopsychosocial model of dementia as a tool to guide clinical practice. Int Psychogeriatr. (2022) 34:7–20. 20561384 10.1017/S1041610210000840

[12] Kitwood T. Dementia reconsidered: The person comes first. Buckingham, England: Open University Press (1997).

[13] Brooker D Latham I. Person-Centred dementia care: Making services better with the VIPS framework. 3rd ed. Routledge, Abingdon, UK: Jessica Kingsley Publishers (2022).

[14] Wang G Marradi C Albayrak A van der Cammen TJ. Co-designing with people with dementia: a scoping review of involving people with dementia in design research. Maturitas. (2020) 139:69–75. doi: 10.1016/j.maturitas.2019.06.003 31351521

[15] Wu B Petrovsky DV Wang J Xu H Zhu Z McConnell ES. Dementia caregiver interventions in Chinese people: a systematic review. J Adv Nurs. (2023) 79:30–41. doi: 10.1111/jan.13865 30264464

[16] Dam AE de Vugt ME van Boxtel MP Verhey FR. Effectiveness of an online social support intervention for caregivers of people with dementia: the study protocol of a randomised controlled trial. Trials. (2021) 22:340. doi: 10.1186/s13063-017-2097-y 28851406 PMC5575867

[17] Cheng ST Li KK Losada A Zhang F Au A Thompson LW. The effectiveness of nonpharmacological interventions for informal dementia caregivers: an updated systematic review and meta-analysis. Psychol Aging. (2020) 35:55–77. doi: 10.1037/pag0000401, PMID: 31985249

